# Multifaceted activities of transcription factor EB in cancer onset and progression

**DOI:** 10.1002/1878-0261.12867

**Published:** 2020-12-23

**Authors:** Elena Astanina, Federico Bussolino, Gabriella Doronzo

**Affiliations:** ^1^ Department of Oncology University of Torino Candiolo Italy; ^2^ Candiolo Cancer Institute‐IRCCS‐FPO Candiolo Italy

**Keywords:** angiogenesis, autophagy, cell‐cycle, lysosome, metabolism, tumor microenvironment

## Abstract

Transcription factor EB (TFEB) represents an emerging player in cancer biology. Together with microphthalmia‐associated transcription factor, transcription factor E3 and transcription factor EC, TFEB belongs to the microphthalmia family of bHLH‐leucine zipper transcription factors that may be implicated in human melanomas, renal and pancreatic cancers. TFEB was originally described as being translocated in a juvenile subset of pediatric renal cell carcinoma; however, whole‐genome sequencing reported that somatic mutations were sporadically found in many different cancers. Besides its oncogenic activity, TFEB controls the autophagy‐lysosomal pathway by recognizing a recurrent motif present in the promoter regions of a set of genes that participate in lysosome biogenesis; furthermore, its dysregulation was found to have a crucial pathogenic role in different tumors by modulating the autophagy process. Other than regulating cancer cell‐autonomous responses, recent findings indicate that TFEB participates in the regulation of cellular functions of the tumor microenvironment. Here, we review the emerging role of TFEB in regulating cancer cell behavior and choreographing tumor–microenvironment interaction. Recognizing TFEB as a hub of network of signals exchanged within the tumor between cancer and stroma cells provides a fresh perspective on the molecular principles of tumor self‐organization, promising to reveal numerous new and potentially druggable vulnerabilities.

## Graphical abstract

Transcription factor EB (TFEB) in stressed cells activates autophagic flux and lysosome biogenesis. Besides this canonical pathway, emerging findings indicate that TFEB regulates cell cycle, metabolism and inflammation. This review summarizes the role of TFEB in cancer onset and progression and advances multiple roles as an oncogene and regulator of the crosstalk circuits between cancer cells and tumor microenvironment (TME).

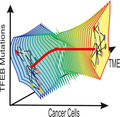

**Abbreviations**
AMPK: AMP‐activated kinase
CDK: cyclin‐dependent kinase
CLEAR: coordinated lysosomal expression and regulation
EMT: epithelial‐mesenchymal transition
ERK: extracellular‐signal‐regulated kinase
GSK: glycogen synthase kinase
HLH: helix‐loop‐helix
MALAT: metastasis‐associated lung adenocarcinoma transcript 1
MAP3K3: mitogen‐activated protein kinase kinase kinase 3
MiT: microphthalmia
PK: protein kinase
Ppar: peroxisome proliferator‐activated receptor
Ppargc: Ppar gamma coactivator
Rheb: Ras homolog enriched in brain
TF: transcription factor
TFEB: transcription factor EB
TGF‐β: transforming growth factor β
TME: tumor microenvironment

## 1 Introduction

In the last 15 years, transcription factors (TF) and enhancers are becoming emergent players in oncogenesis and cancer progression, with a bloom of new information focusing on molecular aberrations and altered regulatory functions resulting in pro‐tumoral genetic landscapes. TF functions are modified in many cancers through direct mechanisms including point mutations, translocations, amplifications, deletions and altered expression, or indirectly by mechanisms altering the binding to promoters [1, 2, 3]. For many years, TF were considered undruggable. However, the recent progress in understanding the mechanisms of DNA–protein and protein–protein interactions, the degradation process and the post‐translational modifications of TF as well as the epigenetic control of their expression have enabled the generation of specific inhibitors, and many clinical trials are underway both in solid cancers and in onco‐hematologic diseases [3].

Here, we illustrate the role of TFEB in tumor biology, envisaging that the control of its de‐regulated activities observed in some cancers could be of therapeutic interest. TFEB belongs to the microphthalmia (MiT) family of basic helix‐loop‐helix (bHLH)‐leucine zipper TF, which includes microphtalmia‐associated TF (MITF), TFE3 and TFEC. It is considered a master regulator of lysosomal and autophagosomal biogenesis and represents a molecular tool to adapt cells to stress, including starvation and energy depletion. However, recent findings clearly demonstrate wider regulatory activities encompassing metabolism, immunity, angiogenesis and inflammation, which are not necessarily connected with autophagy.

## 2 The molecular features of TFEB

**Fig. 1:**
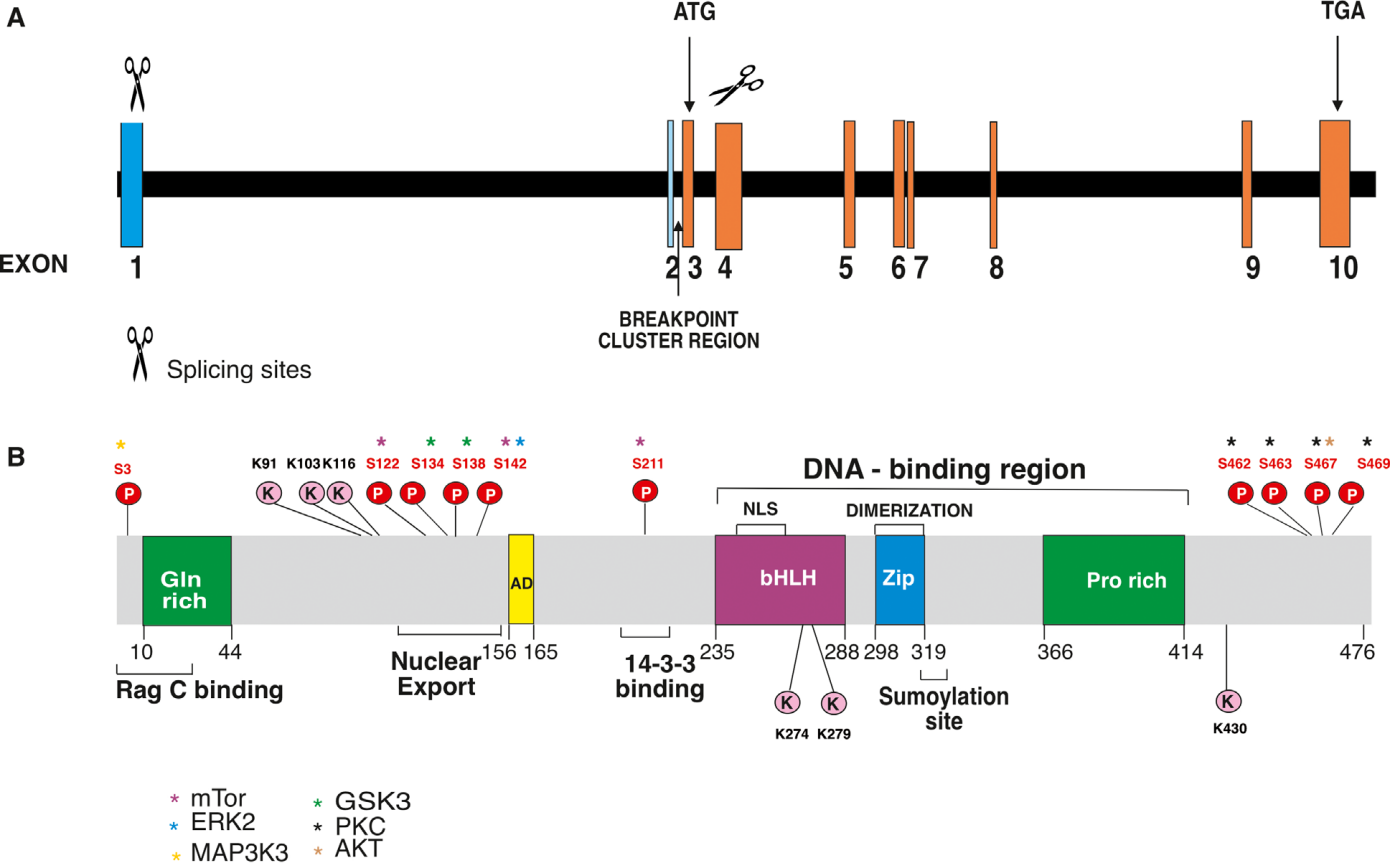
Schematic representation of *TFEB* gene (A) and protein with the relevant domains, regions and amino acid residues undergoing post‐translational modifications (B). (A) Numbers indicate exons; ATG and TGA stop codon at exon 10 and the breakpoint cluster region are shown. (B) AD, activation domain; Zip, leucine zipper domain.

Transcription factor EB was cloned in 1990 and identified as a protein characterized by an HLH and a leucine zipper domain flanked by an upstream basic region, able to recognize an E‐box sequence (CAYGTG) in the heavy‐chain Ig enhancer and in major late promoter of adenovirus [4, 5] as well as in other targeted genes [6, 7]. TFEB structure also contains an acidic and a proline‐rich region (Fig. 1)

A palindromic consensus sequence overlapping that of the E‐Box, called the coordinated lysosomal expression and regulation motif (GTCACGTGAC; CLEAR), was recently described as a common determinant of lysosomal gene promoters regulated by TFEB [6, 8, 9] and has stimulated a large number of studies supporting the concept that TFEB orchestrates autophagy, lysosome functions and is a potential therapeutic target in lysosome storage diseases [10, 11].

Human *TFEB* is located on chromosome 6 (6p21.1) and encodes a 2364‐bp messenger (m) RNA transcript, consisting of two non‐coding and eight coding exons, with a 302‐bp 5′ UTR followed by a start codon in exon 3 and a stop codon in exon 10, followed by a 621‐bp 3′ UTR. A least seven different mRNA that contain alternative 5′ exons have been described with differential and restricted tissue distributions [12]. TFEB is highly conserved during the evolution and present in worms (*Caenorhabidis elegans*), flies (*Drosophila melanogaster*), fishes (*Danio rerio*), amphibian (*Xenopus tropicalis*), avians (*Gallus gallus*) and mammals [13, 14, 15, 16] (Fig. 1). Efficient DNA binding requires its homodimerization or the formation of a heterodimer with TFE3 and MITF [5, 17, 18]. However, the biological meaning of homo‐ and heterodimers is still unknown. The evident homology sequence of TFEB with MITF/TFE and TFE3 predicts a conserved activation domain that is important for the transcriptional activation and is able to bind p300 [19].

## 3 The regulatory mechanisms of TFEB nuclear‐cytosolic shuttling

Transcription factor EB is a cytosolic protein which translocates to the nucleus to trigger specific genetic programs. TFEB nuclear‐cytosolic‐shuttling as well as its nuclear activity are regulated by post‐translational modifications including phosphorylation/dephosphorylation [9, 28], acetylation/deacetylation [29, 30, 31] and sumoylation [32] events.

### 3.1 Phosphorylation‐dependent TFEB cytosolic retention

The most defined system phosphorylating TFEB and thus halting its nuclear translocation is represented by mTORC1 [20, 21, 23, 25] and Rag GTPases, which determine the localization of mTORC1 and TFEB itself on the cytosolic surface of lysosomes [21, 33, 34, 35]. The first 30 amino acid residues of TFEB structure represent the Rag binding site, and its deletion or S3A/R4A point mutations force the localization of TFEB in the nucleus [35].

**Fig. 2:**
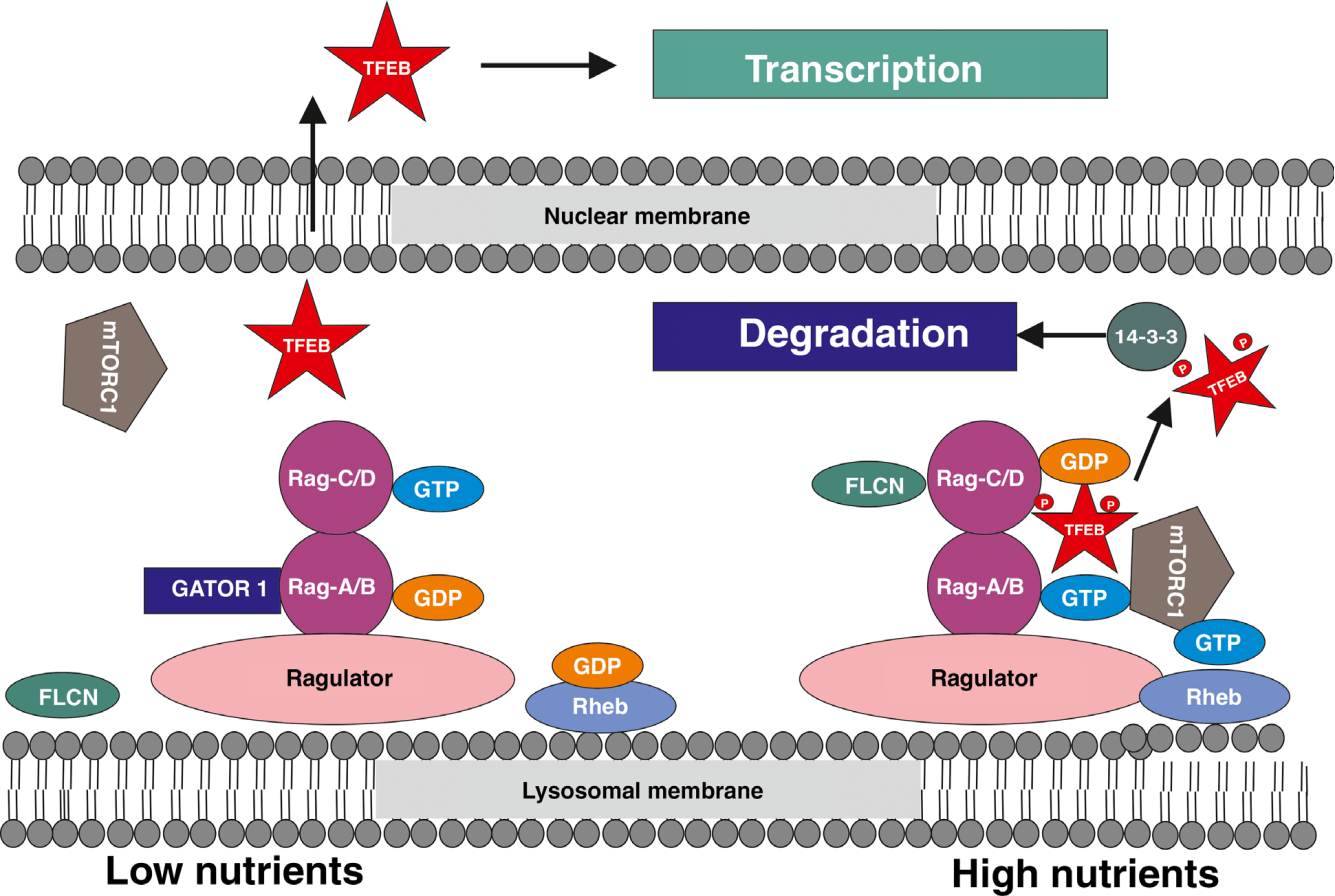
Regulation of the nuclear translocation of TFEB by mTORC1. (Left) With few nutrients, TFEB is not phosphorylated by mTORC1 and is free to undergo nuclear translocation. (Right) In the presence of nutrients and, in particular, amino acids, TFEB is recruited on lysosomal surfaces and phosphorylated by active mTORC1. Phosphorylation of Ser211 allows the binding to 14‐3‐3 protein, which blocks its nuclear entry. TFEB is then degraded by proteasome. For details see text (section 3).

The mTORC1 localizes on lysosomes through a hetero‐complex constituted by Ragulator, a pentameric complex constituted of Lamtor 1–5 with guanidine nucleotide exchange factor activity, and the Rag GTPases, which function as heterodimers [36, 37]. Rag heterodimers consist of two functionally equivalent pairs, RagA or RagB in complex with RagC or RagD. Nutrients trigger the transition from the ‘inactive’ combination of GDP‐bound Rag A and GTP‐bound Rag C (RagA^GDP^:RagC^GTP^) to the ‘active’ RagA^GTP^:RagC^GDP^ state. The ‘active’ Rag heterodimer binds directly to mTORC1 and recruits it to the lysosome, enabling its subsequent activation, which is strongly enhanced by the binding of mTORC1 to the GTP‐bound form of ‘Ras homolog enriched in brain’ (Rheb) GTPase. Two GTPase‐activating protein complexes mediate, in part, the conversion between ‘active’ and ‘inactive’ Rag GTPase states. When nutrients are low, GATOR1 promotes GTP hydrolysis of RagA or B [38]. Conversely, nucleotide hydrolysis on RagC or D is stimulated by folliculin (FLCN), in complex with FLCN‐interacting protein 1 or 2 [33, 39]. In the absence of amino acids, the Rags become inactive (GDP‐bound RagA/B and GTP‐bound RagC/D) and mTORC1 is released again in the cytosol (Fig. 2).

It is likely that a similar scenario occurs with TFEB, as recently detailed by the demonstration that the N‐terminal region is involved in the binding to Rags and relies on the nucleotide active binding configuration, as reported for mTORC1 [35, 40]. In particular, a complex constituted of Raptor, TFEB, Rag and mTOR is required for the TFEB phosphorylation and depends on a direct interaction between TFEB and the Rags in active form [33, 35, 40] (Fig. 2).

Active mTOR on lysosomal surface phosphorylates TFEB at residues S122, S142 and S211. Once phosphorylation of S211 occurs, TFEB is released from the lysosomal surface and is bound by 14–3–3 scaffold protein, which renders TFEB inactive in the cytosol [9, 20, 21, 23, 41]. Phosphorylated S142 and S211 induce the degradation of TFEB through ubiquitin‐proteasome pathway [42], whereas S122 does not modify TFEB subcellular localization but enhances the effect of phosphorylated S211 [23]. Of note and differently from other substrates, the mTOR‐mediated TFEB phosphorylation is independent of Rheb GTPase but just requires Rags [40] (Fig. 1; Table 1).

**Table 1:** Role of phosphorylated serine residues in TFEB cellular localization.

Residue	PK	Cytosolic retention	Nuclear translocation	Nuclear export	Other
3	MAP3K3	No effect	No effect	No effect	Inhibits S211 phosphorylation by mTOR
122	mTOR	No effect	No effect	No effect	Enhancement of phosphorylated S211 effect
134	GSK3β	Yes	No effect	No effect	
138	GSK3β	Yes	No effect	Yes	
142	mTOR	Yes	No effect	Yes	
142	ERK 2	Yes	No effect	Yes	
142	CDK4	No effect	No effect	Yes	
211	mTOR	Yes	No effect	No effect	14‐3‐3 binding
461	PKCβ	No effect	No effect	No effect	TFEB stabilization
462	mTOR	No effect	Yes	No effect	
462	PKCβ	No effect	No effect	No effect	TFEB stabilization
463	mTOR	No effect	Yes	No effect	
466	mTOR	No effect	Yes	No effect	
466	PKCβ	No effect	No effect	No effect	TFEB stabilization
467	AKT	Yes	No effect	No effect	
467	mTOR	No effect	Yes	No effect	
468	PKCβ	No effect	No effect	No effect	TFEB stabilization
469	mTOR	No effect	Yes	No effect	

However, the regulatory role of mTOR on TFEB nuclear localization is likely more complex. In fact, the substitution of S462, S463, S466, S467 and 469 with phosphomimetic aspartate residues forces TFEB nuclear translocation [43] (Fig. 1).

Besides mTOR, other serine/threonine kinases recognize TFEB as substrate and modulate its localization but the biological meaning of these modifications are still poorly understood. Phosphorylation of TFEB S142 by extracellular‐signal‐regulated kinase (ERK) 2 inhibits the nuclear entry of TFEB, and ERK inhibitors induce its nuclear translocation [9, 21]. In osteoclasts, the phosphorylation of S461, S462, S466 and S468 residues by protein kinase (PK) Cβ activated by Receptor activator of nuclear factor kappa‐Β ligand stabilizes TFEB without affecting its subcellular localization, thus suggesting a cooperative mechanism with mTOR [24]. Mitogen‐activated PK kinase kinase 3 (MAP3K3) physically associates with TFEB and phosphorylates S3 residue. This post‐translational modification induced by an abundance of amino acids appears necessary to inhibit the TFEB phosphorylation at residue S211 by mTORC1 [27]. AKT phosphorylates TFEB on S467, contributing to its cytosolic retention as confirmed by the nuclear localization of TFEB S467A mutant [25]. Finally, TFEB S134 and S138 residues can have inhibitory functions when phosphorylated by glycogen synthase kinase (GSK)3β, whereas GSK3 inhibitors favor TFEB nuclear translocation [22] (Fig. 1).

A mirrored mechanism is played by dephosphorylating mechanisms. Lysosomal calcium release through the calcium channel Mucolipin 1 activates phosphatase calcineurin, which binds TFEB dephosphorylating S211 and S142, thus inducing its nuclear translocation [26]. Protein phosphatase 2A activated by oxidative stress [28] dephosphorylates TFEB at S109, S114, S122 and S211 residues, indicating that TFEB may participate in the cellular response to the oxidative stress in a mTORC1‐independent fashion.

Lastly, even in the absence of the demonstration of a direct regulation of TFEB trafficking by AMP‐activated kinase (AMPK), it was reported that the pharmacological activation of this kinase in *C. elegans* and in macrophages challenged with *Staphylococcus aureus* resulted in the nuclear translocation and activation of TFEB that was abolished in AMPK null fibroblasts [44]. AMPK was thought to activate TFEB by inhibiting mTORC1 [45], but pathogen‐induced TFEB activation is likely independent from mTORC1 [46].

### 3.2 Phosphorylation‐dependent TFEB nuclear export

When the stressing conditions cease, the TFEB‐mediated transcriptional activity stops by signals promoting the export from the nucleus to the cytosol (Table 1). A hydrophobic nuclear export sequence has been mapped at residues 129–152 and encompasses S142 and S138, which may be phosphorylated by mTOR or ERK2 [9, 20, 21, 23, 41] and by GSK3β [22]. Interestingly, the phosphorylation of S142 by mTor or ERK2 primes the nuclear export sequence to be phosphorylated by GSK3β at S138, an efficient nuclear export [47, 48] (Fig. 1; Table 1).

However, new findings indicate a more complex picture of the TFEB regulation by phosphorylation events. For instance, during glucose limitation but not in the absence of amino acids, mTORC2 inactivates GSK3β through AKT and inhibits TFEB nuclear export [47]. These phosphorylating steps allow TFEB to be shuttled to the cytosol through exportin‐1 [47, 48, 49]. Another example is the effect of Cyclin‐Dependent Kinase (CDK) 4, which is under the direct transcriptional control of TFEB [7]. TFEB/CDK4 nuclear co‐localization when mTOR was pharmacologically blocked by Torin‐1 has recently been described. Under these conditions, CDK4 phosphorylated TFEB at residues Ser142, allowing its nuclear export [50] (Table 1).

### 3.3 The role of acetylation/deacetylation process in TFEB‐induced transcription

New and partially conflicting findings have shown that TFEB function is also regulated by its acetylation and deacetylation. However, these effects likely differ with respect to the cellular context. In microglia, the deacetylation of TFEB significantly improves autophagy and lysosomal function. This process is mediated by nuclear SIRT1, which binds and deacetylates TFEB at K116 residue, thus increasing its transcriptional function [31]. In cancer cell lines (HCT116, HEK293T) inhibitors of histone deacetylase increase TFEB activity by favoring acetylation of K91, K103, K116 and K430 at nuclear level [29]. The histone acetyltransferase ‘General control non‐repressed protein 5’ has been reported to inhibit lysosome biogenesis by acetylating TFEB at K116, K274 and K279 residues and suppressing its transcriptional activity. Mechanically, the acetylation process inhibits TFEB dimerization and the capability to bind the promoter of target genes [30]. Finally, it has been reported that histone deacetylase is able to bind *TFEB* promoter to inhibit its expression [51] (Fig. 1).

### 3.4 TFEB sumoylation

It has been reported that TFEB contains the sumoylation consensus sequence ΨK*X*E and undergoes in vivo this post‐translational modification. However, the impact of this process in TFEB biology is still unknown [32] (Fig. 1).

## 4 TFEB‐mediated genetic programs involved in cancer progression

**Fig. 3:**
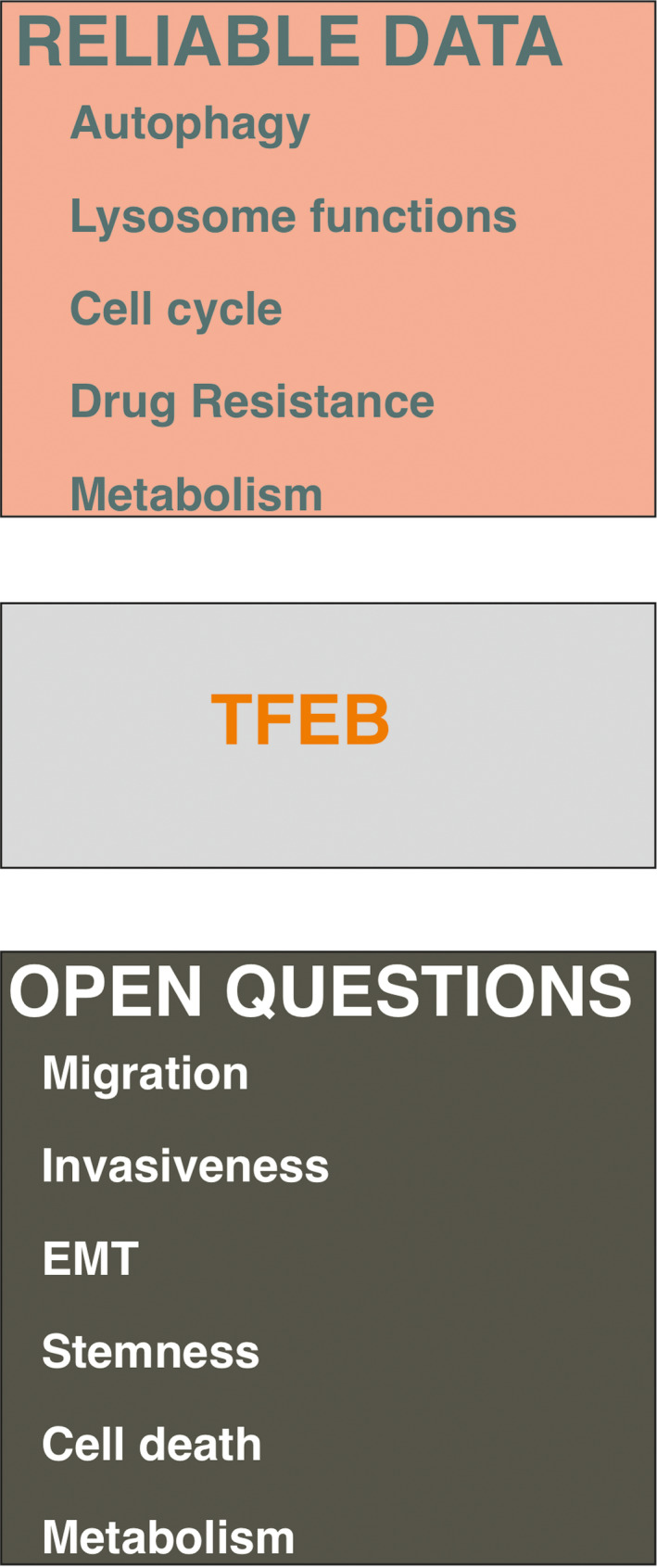
Established and putative activities of TFEB in cancer cells. Besides being mutated in a subset of renal carcinomas, TFEB influences biological activities, which has a significant impact on the behavior of solid cancers including pancreatic and renal carcinomas and melanomas. However, in many areas of cancer biology the role of TFEB is largely unknown, with potential new areas of investigations.

**Fig. 4:**
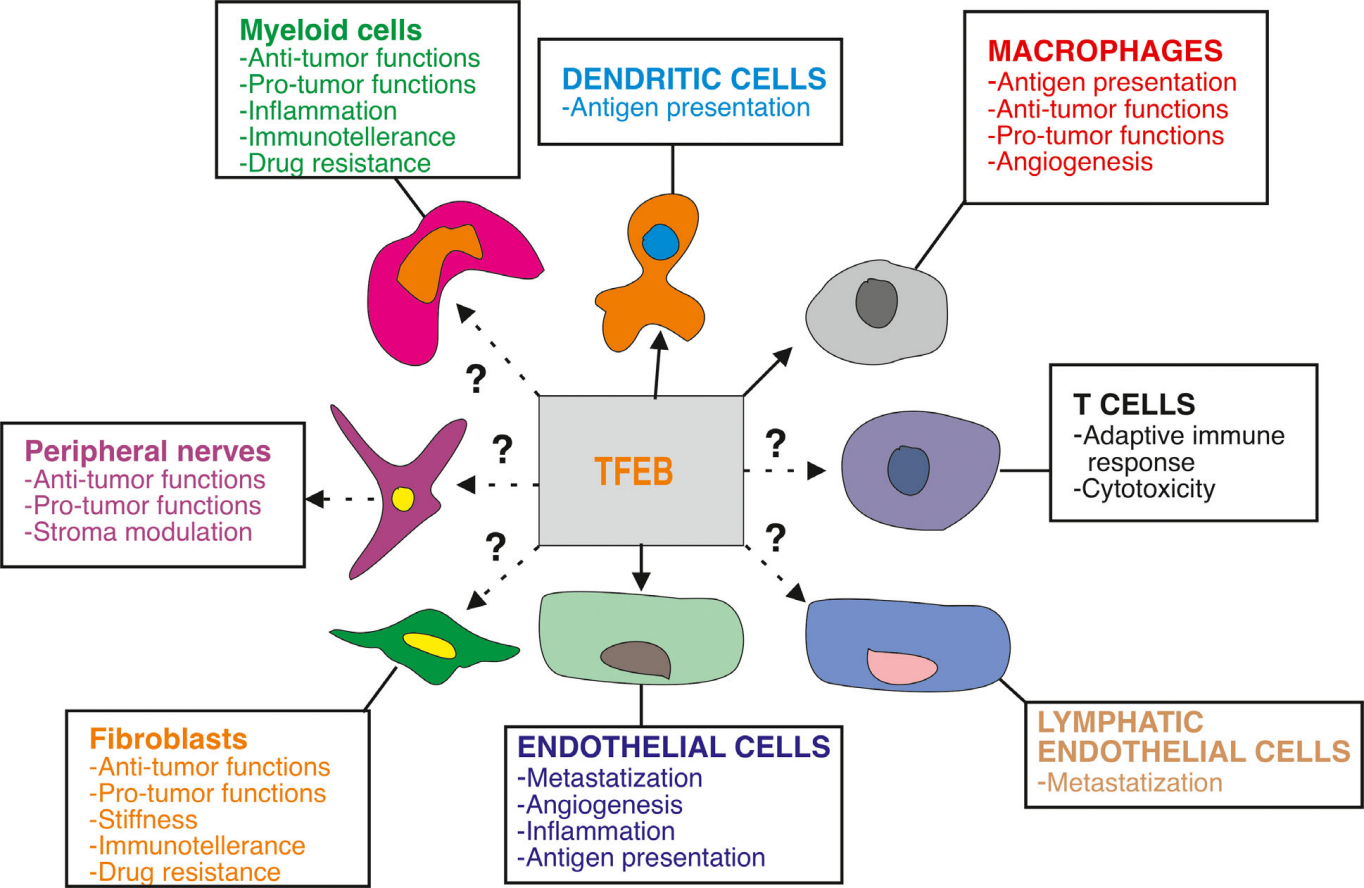
Established and putative activities of TFEB in TME. Relevant cell targets and their biological functions relevant for cancer progression that can be influenced by TFAB activities. Arrows and dotted arrows respectively indicate the presence of direct or indirect experimental data supporting the role of TFEB in the indicated biological functions.

Cancer onset and progression rely on genetic alterations that are largely somatic, but the cancer success is strictly conditioned by the features of tumor microenvironment (TME), which may favor or halt the expansion of cancer cells and condition the onset of resistance to the therapies. Here we illustrate established and emerging transcriptional programs orchestrated by TFEB that may influence both cancer and TME cells (Figs 3 and 4).

### 4.1 Lysosome biogenesis and autophagy

Seminal papers of Ballabio’s group were the first to demonstrate that TFEB promoted the expression of more than 400 genes connected with the biology of lysosomes [11]. These genes encode for proteins that orchestrate the expression, localization, entrance, influx and performance of lysosomal and non‐lysosomal enzymes participating in the destruction of cellular macromolecules such as proteins, glycosaminoglycans, lipids, glycogen and hemoglobin [6, 8, 9]. In addition, TFEB can promote lysosomal exocytosis, allowing their cargo secretion through the fusion to cell membrane [26]. In the CLEAR network there are some genes that are known to play a direct role in the different steps of autophagy (*ATG9, UVRAG, VPS11, VPS18, WIPI*) [9].

These findings have been immediately connected with the instrumental role of lysosomes in the ‘lysosomal storage diseases’ and in the establishment of autophagic flux [52]. At present, TFEB may be considered one of the most important principle regulators of autophagy which has opposing, context‐dependent roles in cancer. The role of autophagy in cancer has been extensively reviewed elsewhere, which we refer to. Two opposite scenarios may be envisaged. Failure of autophagy interferes with oncogene‐induced senescence and thereby might favor uncontrolled proliferation of cancer cells. Conversely, in established cancers, autophagy is required for cell survival by providing nutrients [53, 54, 55, 56]. Accordingly, specific correlations between the level of TFEB and the autophagic flux have been described in several cancers (breast, pancreas, lung and in part discussed in section 5 [57, 58, 59, 60].

### 4.2 Cell cycle

**Fig. 5:**
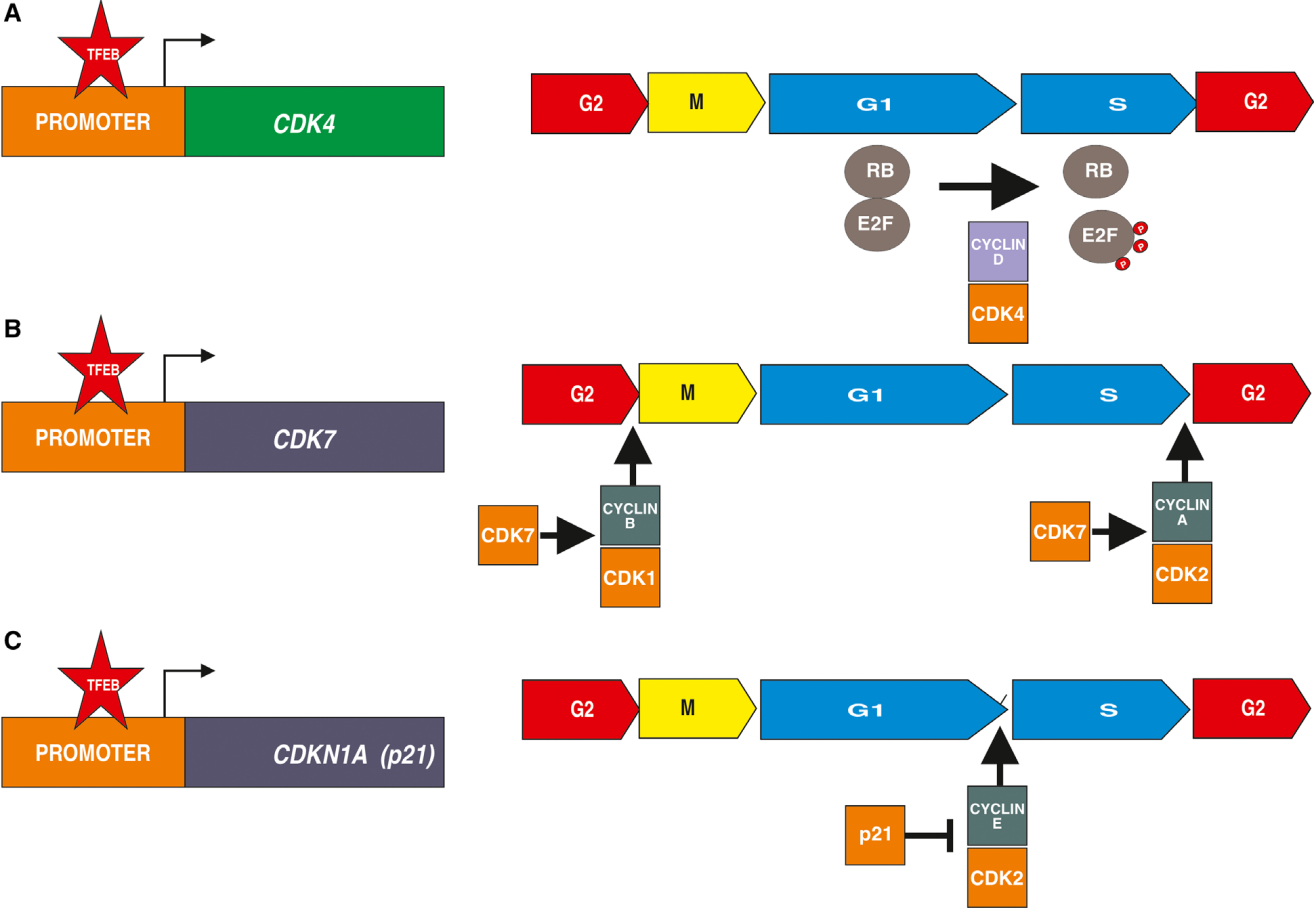
Effect of TFEB on cell cycle. TFEB directly control the transcription of *CDK4* (Panel A), *CDK7* (Panel B) and *CDKN1A* (Panel C) resulting in a balance between activators and inhibitors of the cell cycle. The final effect is the regulatory contribution of TFEB on G/S, G2/M and S/G2 phases of cell proliferation. Panel A: TFEB promotes the expression of *CDK4* resulting in the phosphorylation of retinoblastoma protein (Rb) and the nuclear translocation of E2F to transcript genes necessary of S‐phase. Panel B: TFEB promotes the expression of *CDK7*, which participate to the formation of CDK activating kinase complex. This complex is required to the activation of CDK1 and CDK2 respectively resulting in the regulation of G2/M and S/G2 phases. Panel C: TFEB promotes the expression of cell cycle inhibitor p21 (*CDKN1A*), which blocks the progression G1/S, by inhibiting cyclin‐dependent kinase activities.

A first suggestion that TFEB regulates the cell cycle was provided in cancer cell lines lacking TFEB and TFE3 and treated with the genotoxic agent etoposide. In this model, the knock‐out of these TF resulted in reduced expression of multiple genes implicated in cell cycle progression as well as genes involved in chromosome segregation and cytokinesis [61]. These data were confirmed in a model of triple negative breast cancer, in which *TFEB* silencing combined with doxorubicin resulted in a down‐modulation of cell‐cycle genes. These observations were refined in TFEB‐deleted endothelial cells [7] and hepatoblasts [62], which showed a block of the G1–S cycle transition. In endothelial cells, TFEB bound (*CDK4*) promoter and in the absence of TFEB, its transcriptional rate was impaired. As a consequence the phosphorylation of Retinoblastoma protein is reduced, blocking the nuclear translocation of E2F to transcript genes necessary for S‐phase [7]. Similarly, in HeLa cells, TFEB deletion resulted in reduced Rb phosphorylation and the TFEBS142A active mutant increased the expression of *CDK4* and *CDK7* [61]. The direct transcriptional control of *CDK4* by TFEB, together with the recent observation that TFEB Ser142 residue is a substrate of CDK4 itself [50], opens new perspectives in the co‐regulation of lysosome biogenesis and cell cycle (Fig. 5).

Besides inhibiting CDK4, TFEB depletion reduces the expression level of p21 cyclin kinase inhibitor, while its overexpression has an opposite effect through a direct binding to its promoter in a p53‐dependent manner [63]. Doxorubicin‐mediated DNA damage promoted the activation of *CDKN1A* (CDK Inhibitor 1A; p21) through TFEB, leading to cell cycle arrest [63]. Of note, a positive regulatory loop between p53 and TFEB has been described. In HeLa cell p53 activated TFEB via the up‐regulation of Sesn1 and Sesn2, which inhibits mTORC1 signaling. In parallel, TFEB further enhanced p53 stabilization [61] (Fig. 5).

A further link between cell cycle, TFEB activity and autophagy has been recently demonstrated in a cellular model, in which CDK inhibitor 1B (p27) was deleted. This study demonstrated that p27 was recruited to lysosomes to interact with LAMTOR1, a component of the Ragulator complex required for mTORC1 activation. Binding of p27 to LAMTOR1 prevented Ragulator assembly and mTORC1 activation, promoting autophagy in a TFEB‐dependent manner [64].

Another control of cell proliferation by TFEB relies on apoptosis. TFEB knockdown induced cell death and the decrease of cell viability via the up‐regulation of pro‐apoptotic molecules belonging to interferon and tumor necrosis factor pathways and the down‐modulation of apoptosis inhibitors [65].

### 4.3 Metabolism

In cancer, malignant cells show a high metabolic flexibility to adapt themselves in response to cell‐extrinsic and cell‐intrinsic cues. According to the role of TFEB in regulating autophagy and the diverse substrates that can be degraded through this process, it is not surprising that TFEB has the potential to participate indirectly with nearly all aspects of carbon metabolism, including mitochondrial activities [66]. However, direct and specific effects of TFEB on lipid metabolism have been described. TFEB recognizes binding sites on the promoters of Peroxisome proliferator‐activated receptor (Ppar) gamma coactivator 1α (*P* *PARGC*) and Ppar γ2 (*P* *PARG2*), which are regulators of lipid anabolic and catabolic pathways [67, 68]. When overexpressed in the liver, TFEB increases the expression of *PPARGC1α*, which fosters the β‐oxidation of fatty acids and the ketogenesis. Conversely, TFEB reduces the expression of enzymes involved in some lipid biosynthetic pathways [69]. However, in mice fed with western diet, TFEB overexpression has been reported to upregulate enzymes involved in cholesterol and bile acid synthesis [70]. In adipose tissue, TFEB expression stimulated by glucose and insulin is necessary to sustain adipocyte differentiation by a mechanism mediated by Pparγ2 [71], which promotes lipogenesis [72]. In cardiomyocytes, TFEB deletion reduces their ability to oxidize fatty acids and shifts the energetic metabolism towards the use of glucose [73]. Finally, emerging findings demonstrate that TFEB is involved in cAMP‐induced lipolysis regulated by calcium influx mediated by the store‐operated calcium entry [74].

Besides controlling lipid metabolism, in active skeletal muscle, TFEB controls energy expenditure and mitochondrial activity in an autophagy‐ and Ppargc1‐independent manner. Overexpression of *TFEB* induces mitochondrial biogenesis and improves oxidative phosphorylation. These effects are associated to direct control of TFEB of glucose homeostasis through *GLUT1/4* expression and insulin sensitivity via nitric oxide synthase [75].

Therefore, it is intriguing to speculate that the effect of TFEB on metabolism might depend on specific tissue commitments and defined metabolic conditions. This hypothesis is further sustained by the control of *TFEB* expression by the cAMP response element‐binding protein [76], which regulates lipid and glucose metabolism.

### 4.4 Epithelial‐mesenchymal transition

Epithelial‐mesenchymal transition (EMT) has a key role in neoplastic transformation and in the metastatic process [77]. The role of TFEB in establishing the equilibrium between epithelial and mesenchymal phenotypes was discovered in 2005 [78] and has not been studied in depth since. *TFEB* overexpressed in 3T3 and mouse embryonic fibroblasts directly activated E‐cadherin promoter, and TFEB was shown to be required for E‐cadherin endogenous expression in these cells. TFEB also upregulated WT1 [78], a TF able to regulate EMT in both directions depending on context [79] and decreased the expression of EMT regulator Snail. Conversely, E‐cadherin expression in epithelial cell lines did not depend on TFEB. Moreover, *TFEB* overexpression led to E‐cadherin downregulation. Taking into consideration EMT as a milestone of cancer development, it would be worth investigating whether TFEB is involved.

## 5 TFEB and cancer subtypes

Genetic alterations of TFEB are mainly involved in the pathogenesis of tumors developing in kidney, exocrine pancreas and melanomas. Besides these cancers, TFEB alterations have been described in colorectal cancer [80, 81], gastric carcinoma [82], non‐small cell lung cancer [59] and breast cancer [83]

### 5.1 Renal carcinomas

Gene fusions involving two members of the MiT family, TFEB and TFE3, characterize a subset of sporadic clear cell renal carcinomas often characterized by expression of cathepsin k and melanocytic markers and defined as MiT family translocation renal cell carcinoma [84]. The most common variant results from the fusion between TFEB and the non‐protein encoding Metastasis associated lung adenocarcinoma transcript 1 gene (*MALAT1*) on chromosome 11q13. Fusion occurs within a breakpoint cluster region upstream of *TFEB* exon 3 or 4 and within a 1205‐bp breakpoint region of *MALAT1* [85, 86, 87]. This translocation results in the substitution of *TFEB* promoter and a consistent increase of TFEB protein level able to translocate into the nucleus [85, 86]. When overexpressed in renal carcinoma cell lines, *MALAT1‐TFEB* increases cell proliferation, invasiveness and in vivo tumorigenicity [88]. Single cases with different fusion patterns have been described and include KHDRBS2, COL21A1, CADM2, CLTC and EWSR1 [87].

The pathogenetic role of the overexpression of TFEB in this subset of clear cell renal carcinomas has been recently supported by the analysis of transgenic mice in which *TFEB* was specifically expressed in kidney under the control of cadherin 16 promoter. These mice developed tubular cysts morphologically similar to those observed in MALAT1‐TFEB clear cell renal carcinomas, and then showed metastatic cancer. This process was autophagy‐independent and characterized by activation of Wnt pathway

Interestingly, a recent report in gastric carcinomas extends this observation, showing that TFEB activated Wnt/β‐catenin signaling to initiate a pro‐invasive program [82]. The crosstalk between TFEB and Wnt/β‐catenin pathways is emerging as a relevant and probably more general mechanism of oncogenesis [90]. The molecular candidate to bridge these pathways is GSKβ, a regulator of TFEB nuclear trafficking [22, 91] which belongs to the Wnt degradation complex [92]. It has been reported that TFEB inhibition in AMPK null mice resulted in impairment of endodermal differentiation. The compromised endolysosomal system resulting from TFEB inactivation blunted Wnt pathway [45]. It is intriguing to speculate that upon Wnt activation, components of Wnt degradation complex, including GSKβ, are sequestered into multivesicular bodies [92] enabling TFEB stabilization and nuclear activity.

Transcription factor EB is also a determinant effector of Birt–Hogg–Dubé syndrome, a genetic disease caused by germ line mutations in the RagC and RagD activator FLCN and characterized by benign skin tumors, lung and kidney cysts and renal cell carcinoma [93]. Mice with kidney‐specific knockout of *Flcn* developed polycystic disease and pre‐neoplastic foci with increased nuclear localization of TFEB. This phenotype reverted when *Flcn* null mice were backcrossed with mice lacking renal *Tfeb* [40].

The oncogenic role of overexpressed *TFEB* may also result from its amplification and in the last decade many cases of renal carcinomas with amplified *TFEB* have been reported [94]. Of note, in some cases *TFEB* was co‐amplified with *VEGFA* [95], known to be a pivotal player in tumor angiogenesis and to be amplified in some aggressive types of colorectal carcinoma.

TFEB has been also implicated in the mechanisms of acquired resistance to mTOR inhibitors, which are exploited in the treatment of metastatic renal carcinomas. The inhibition of mTOR led to enhanced TFEB nuclear translocation and *CD274* (Programmed Death‐Ligand 1; PD‐L1) expression by a direct activation of its promoter, resulting in an immune evasion via suppression of the cytotoxic function of CD8^+^ cells [96]

### 5.2 Pancreatic carcinomas

Pancreatic adenocarcinomas show unique and specific features dependent on an extensive and prominent stromal reaction, resulting in a hypovascular and hypoxic TME, reprogramming of cellular metabolism and evasion of tumor immunity [97].

Recently, increased mRNA and protein expression of TFEB, MITF and TFE3 was detected in pancreatic ductal adenocarcinoma (PDAC) cell lines and patient cancers resulting in increased autophagic flux [57, 98], which increases amino acid availability exploited for tumor growth. Interestingly, this subset of tumors is characterized by the failure to traffic these TF because of an aberrant expression of importin 8 [57], which regulates direct nuclear import of specific cargos. Furthermore, reduction of *TFEB* expression impairs PANC1 anchorage‐independent growth, indicating its relevant role in pancreatic oncogenesis [91].

The role of TFEB in sustaining PDAC progression is further supported by studies on micro RNA (miR)29a, which downregulates TFEB through direct interactions with the 3′UTR binding site and reduces autophagy and invasive properties of PDAC cell lines [99]. Interestingly, transforming growth factor β (TGF‐β), which is one of the signaling systems pivotal in PDAC behavior [97], has been demonstrated to activate a TFEB‐mediated autophagic process that favors PDAC cell migration and metastasis [100].

TFEB also seems to be involved in mechanisms of acquired resistance to targeted therapies in PDAC. In in vitro and in vivo PDAC models, MEK inhibitors enhanced lysosome biogenesis in a TFEB‐dependent manner, which resulted in sequestration and inactivation of the inhibitor in the lysosomal compartment. It is likely that genetic depletion of TFEB leads to a decreased lysosomal biogenesis of MEK inhibitors and potentiates tumor sensitivity to MEK inhibition [101].

### 5.3 Melanomas

The MiT gene family includes MITF, which is recognized as a master regulator of melanogenesis and a melanoma oncogene [102]. Increasing evidence indicates a potential role of TFEB in melanoma oncogenesis and a specific regulatory circuit has been described between MITF and TFEB itself. In fact, MITF positively regulates the expression of *TFEB* by a direct control of promoter activity through the binding to intron 1 [103].

Human melanomas are characterized in 40–60% of the cases by the presence of mutated *BRAF* [104]. In *BRAF*‐mutated melanomas, autophagy has been reported to exert both pro‐ and anti‐tumor activity [105, 106]. Furthermore, BRAF inhibitors, which are currently the standard therapeutic regimen in BRAFV600E melanomas, induce autophagy [107]. BRAF^V600E^ has been reported to phosphorylate TFEB by an Erk‐dependent mechanism and inhibit TFEB transcriptional programs. The use of a BRAF inhibitor has been demonstrated to reverse this condition, activate autophagy and reduce in vivo tumor growth. Accordingly, the expression of the dominant active TFEBS142A in A375 melanoma cells increased their in vivo tumorigenic activity. The inhibition of the transcriptional program triggered by TFEB, promoted tumor progression and chemoresistance to BRAF inhibitor, which were associated with TGF‐β‐mediated epithelial–mesenchymal transition [108].

Melanomas are characterized by a high mutational burden, which represents a molecular advantage in the response to immunotherapy strategies. Interestingly, in metastatic melanomas, TFEB is positively correlated with the expression of genes required for the immune response, posing important therapeutic questions about the role of TFEB in immunotherapy [109].

## 6 TFEB and tumor microenvironment

The tumor microenvironment encompasses a heterogeneous population of differentiated and progenitor cells that play a critical role in regulating tumor fate. For example, endothelial cells, their precursors and pericytes are essential for tumor angiogenesis, a process that allows oxygen and nutrient supply, and the new vessels formed contribute to the recruitment of leukocytes and the escape of tumor cells from the primary tumor [110]. Fibroblasts and activated fibroblasts (cancer‐associated fibroblasts) dictate the features of extracellular matrix and its stiffness, support or contrast tumor growth as well as the immune response [111]. The cellular arms of innate and adaptive immunity participate to a complex network of signals, which drive an efficient or inadequate immune response to cancer cells and their invasiveness properties [112, 113]. Peripheral nerves (sympathetic, parasympathetic and sensory) interact with tumor and stromal cells to promote the initiation and progression of a variety of malignancies [114]. Finally, TME strongly influences the response to therapies and can contribute to the acquisition of resistance [115].

### 6.1 Immune system

Transcription factor EB was discovered as a binding protein of the µE3 enhancer of the human immunoglobulin heavy chain locus in human B cells [4]. This finding prefigured a role of TFEB in regulating activities of the immune system and many studies have indicated so far that TFEB has a principal role in regulating aspects of innate immunity conserved during the evolution from worm [44, 116] to human.

In this scenario, the most relevant cellular target of TFEB are phagocytic cells, in particular macrophages, involved in removing pathogens. The effect of TFEB in protecting cells against infective agents is mainly linked to the biogenesis of lysosomes and their role in phagocytosis [44, 46, 117].

TFEB also regulates other macrophage functions more strictly connected with cancer biology independently from the canonical regulation of autophagy and lysosome activities.

Macrophages undergo specific differentiation depending on the local tissue environment and assume distinct functional phenotypes. In TME, macrophages can differ in M1 or M2 phenotypes, which respectively have anti‐ and pro‐tumor activities [118].

Transcription factor EB is probably able to activate both M1‐ or M2‐like macrophages according to the features of inflammatory stimulus [119, 120, 121].

In alveolar‐ and bone marrow‐derived macrophages, the deletion of LAMTOR1, a scaffold protein required to activate mTORC1, results in TFEB activation, and increases autophagy and acquisition of the M2 phenotype. This process is connected with the increased production of interleukin‐6 and tumor necrosis factor‐α by a direct effect of TFEB on their promoters [119]. In a model in which mesenchymal stem cells are co‐cultured with macrophages, the activation of TFEB increases the polarization of M2‐like macrophages by a lysosomal‐dependent mechanism [121].

Tumor‐infiltrating macrophages show the opposite response. In macrophages, TFEB upregulated suppressor of cytokine signaling 3, halting Stat3 activation and thereby blocking M2‐like polarization independently of the stimulated autophagy. Furthermore, TFEB activated the transcription of *PPARG*, which blunts NFκB activation, resulting in downregulation of the inflammatory response [120, 122]. When macrophages are stimulated with conditioned media of different tumor cell types, TFEB is retained in the cytosol with the appearance of markers of M2 polarization. These macrophages show a reduced ability to present the antigen by down‐modulation of major histocompatibility complex (MHC)‐II and the co‐stimulatory molecule CD80. In vivo, macrophages lacking TFEB co‐injected with tumor cells enhance tumor growth with increased infiltration of M2‐like macrophages, reduced infiltration of CD8^+^ cells, and enhanced angiogenesis [120, 122]. Additional insights for the M1‐polarizing effect of TFEB in a tumor context are provided by the anti‐tumor effect of chloroquine, which switches macrophages from the M2 to the M1 phenotype by a TFEB‐dependent mechanism [123].

The TFEB‐regulating effect of lysosome functions is further exploited by antigen‐presenting cells, in particular by dendritic cells. MHC I and II molecules present protein fragments to CD8^+^ and CD4^+^ T cells, respectively, with the final aim to eliminate host antigens. Endogenously synthesized antigens in the cytosol are presented as peptides bound to MHC I molecules, whereas exogenous antigens ingested into endocytic compartments of antigen‐presenting cells are presented as peptides bound to MHC II molecules. In addition to these pathways, exogenous antigens can be processed to MCH class I by cross‐presentation [124]. In dendritic cells, activated TFEB downregulates MHC class I‐restricted antigen cross‐presentation and upregulates the processing and presentation of antigen by MHC class II. Mechanistically, this effect results from the increased expression of lysosomal proteases coupled with increased lysosome acidification [125]. Furthermore, TFEB participates in the recruitment of dendritic cells to the lymph node where antigen presentation occurs. Upon lipopolysaccharide sensing, lysosomal calcium efflux by transient receptor potential cation channel, mucolipin subfamily‐1 (TRPML1) promotes sustained myosin IIA activity at the rear of the dendritic cells and thus stabilizes F‐actin and increased cell migration. Concomitantly, TFEB translocates to the nucleus to maintain a high level of *trpml1* gene expression [126].

The regulatory role of TFEB in antigen presentation to T cells by antigen‐presenting cells could influence the cancer treatment based on immunotherapies. In particular, emerging data indicate a good correlation between the amount of tumor neoantigens and the success of clinical tumor immunotherapies [127].

A role of TFEB in humoral immunity has been envisaged due to its ability to bind *CD40LG* promoter and positively regulate its expression in activated CD4^+^ cells. In this way, TFEB controls the T‐cell‐dependent immunoglobulin response [128].

### 6.2 Vascular system

Although the regulatory activities of TFEB in tumor angiogenesis are still unknown, numerous studies in embryo development and in other pathological conditions support a framework suggesting a role of TFEB in the formation of the tumor vascular bed.

Mouse mutants have highlighted the role of TFEB as a pro‐angiogenic factor both in embryonic [14] and in adult life [7, 129]. Mice carrying a null mutation at the Tfeb locus in a homozygous state are characterized by defective vascularization of the placenta, leading to death of the embryos between E9.5 and E11.5. In wild‐type mice, Tfeb was expressed at very low levels at E8.5‐ to E10.5‐day‐old embryos but highly expressed in labyrinthine trophoblasts from 8.5‐day‐old placentas. In Tfeb null mice, the vascular invasion of labyrinthine trophoblast layer is blocked and capillaries stop in the chorion. Mechanistically, Tfeb null mice expressed a lower level of Vascular Endothelial Growth Factor (VEGFA) in the trophoblast than did wild‐type mice. The placenta hypovascularity results in severe hypoxia, which determines embryo death. This observation has been further refined in endothelial‐specific Tfeb null mouse mutants and in human TFEB‐silenced endothelial cells [7]. In this model, endothelial cells start to express Tfeb at E8.5, which is maintained after birth. Embryonic lethality of mutants is observed at E10.5. The defects of vascular structures rely mainly on an impairment of the vascular remodeling of the primitive vascular plexus. After birth, Tfeb deletion inhibits the retinal vascularization and the maturation of renal glomeruli, with loss of endothelial fenestration and podocyte foot processes. Study of the molecular mechanisms sustaining this phenotype indicates that in endothelial TFEB null mice, the cell cycle is blocked for the reduced expression of CDK4 and other mitotic genes. Furthermore, these mice carry a defect of VEGFR2 functions based on the combined effect of Tfeb deletion on Vascular endothelial growth factor receptor 2 (VEGFR2) trafficking and the post‐transcriptional regulation of its gene expression. TFEB inhibits the expression of Myosin 1C, which delivers VEGFR2 to plasma membrane [130] and inhibits the expression of miRNA‐15a/16‐1 cluster, which specifically targets VEGFR2 3′UTR [131].

During the tumor angiogenic process, autophagy in the blood vessels is emerging as a critical mechanism enabling endothelial cells dynamically to accommodate their higher energy demands to the extracellular environment and connect with other components of the tumor stroma through paracrine signaling [132]. Interestingly, extracellular proteoglycan decorin has been demonstrated to activate endothelial autophagy through TFEB and its nuclear translocation required the catalytic activity of VEGFR2 [133]. This study parallels the observation that VEGF stimulates the expression and activation of TFEB [129].

Transcription factor EB has also been reported to play a role in the angiogenic response after ischemia. Endothelial overexpression of *TFEB* improves blood perfusion and increases capillary density after hindlimb ischemia in mouse. The TFEB pro‐angiogenic effect is mediated by the significantly increased expression of autophagy genes and by the activation of AMPK signaling [129].

The role of capillaries in tumor progression is not just limited to providing nutrients and oxygen.

In the metastatic cascade, the interactions of the metastatic cancer cell with the vascular wall in the primary tumors and in distant organs is largely intuitive. In the primary tumor, the capillaries are poorly structured and leaky, thus facilitating the intravasation of cancer cells to the bloodstream. In contrast, the extravasation in a secondary tissue is highly selective and is considered a limiting step of the metastatic process [134]. Many studies indicate that metastatic cancer cells actively adhere to the endothelial surface and then pass through the cell homotypic junction, similar to that observed during leukocyte diapedesis in inflammatory processes [135]. Recently, some studies have drawn attention to the endothelial anti‐inflammatory role of TFEB [136, 137]. TFEB activation in endothelial cells results in the reduction of the synthesis of inflammatory cytokines and adhesive molecules and the adhesion of circulating monocytes. These effects are independent of the autophagic flux but rely on the suppressor activity on the Nuclear factor kappa‐light‐chain‐enhancer of activated B cells (NF‐κB) pathway. TFEB suppressed IκB kinase activity to protect IκB‐α from degradation, leading to reduced p65 nuclear translocation [136]. Furthermore, the overexpression of *TFEB* in endothelial cells activates the production of anti‐oxidant species by direct binding on the promoter of heme oxygenase‐1 and superoxide dismutase 2 [137].

### 6.3 Peripheral nervous system

In the same way that increased blood vessel formation is necessary for tumor growth, nerve density nearly doubles in tumors compared with non‐neoplastic tissue controls and with the increase in nerve density correlated with aggressiveness of many solid tumors, including prostate, colon, head and neck, pancreas stomach and lung [138, 139, 140]. The effect of peripheral nervous system stimulation on cancer is largely unknown but the data available indicate that adrenergic and sensory stimuli exert pro‐tumoral activity, whereas cholinergic signals exhibit tissue‐dependent effects. The overall effect of peripheral nervous stimulation on cancer progression depends on a direct modulation of both cancer and stroma cells [114]. The role of TFEB in the modulation of cancer peripheral nervous system is mostly speculative. Of note, TFEB has been demonstrated to regulate the formation of myelin [141, 142], which is instrumental in perineural invasion and tumor spread [114, 143].

## 7 Conclusions

The emerging results about the molecular and biological activities of TFEB envisage relevant functions in a wide array of cancers. However, TFEB therapeutic targeting is dependent on a detailed understanding of the mechanistic complexities that govern TFEB activities and several issues have to be faced and solved. First, understanding the impact of deregulated kinase cascades described in many cancers on the phosphorylating events involved in TFEB regulation may help pinpoint possible interactions between small kinase inhibitors with drugs that modulate this TF [144].

Secondly, TFEB represents a node of an undefined TF network, which drives specific and context‐dependent transcriptional programs. So far, little information (Table 2) is available on TF‐regulating *TFEB* expression, as well as on those TF genes regulated by TFEB. These interactions pose the question of the genetic programs orchestrated or modulated by TFEB, besides the so far well‐established canonical pathway sustaining autophagy. Furthermore, it might be relevant to describe an association between the level of TFEB activation and the type of transcriptional response. A paradigmatic example is provided by the hypoxia‐inducible factor, which determines the repertoire of gene expression according to the severity of hypoxic conditions [145]. Attempts to put TFEB in a defined transcriptional cellular landscape are mandatory to understand how the different and in part unrelated biological activities described in this review (e.g. the role of TFEB in immune system) participate in a distinctive and final cell outcome. Furthermore, a better description of TFEB‐TF loops is required to predict limits and chances of TFEB targeting.

**Table 2:** Regulatory circuits between TFEB and other TF.

TF	References	Notes
TF expression regulated by TFEB^a^		
ATF4	[146]	During osteoblast differentiation, TFEB regulates its expression by an unknown mechanism
DDIT3	[146]	During osteoblast differentiation, TFEB regulates its expression by an unknown mechanism
WT1	[78]	TFEB upregulates WT1 in kidney. Mechanism was not analyzed
TP53	[61]	TFEB increases p53 protein stability by an unknown mechanisms
TF regulating TFEB expression		
AR	[147]	AR upregulates *TFEB* transcription through binding 2 androgen response site on *TFEB* promoter
CREB	[76]	Direct binding to *Tfeb* promoter in the liver
ETS2	[148]	Binds *TFEB* promoter under oxidative stress
KLF2	[136]	Binds *TFEB* promoter and increases its expression
MYC	[51]	Binds *TFEB* promoter and reduces its expression
PPARA	[149]	Direct binding to and up‐regulation of *Tfeb* promoter in the liver
PPARG	[71]	In mouse adipocytes, Tfeb binds and activates *Pparg* promoter. This activity is increased by TFE binding to the promoter.
TFEB	[9]	TFEB drives the expression of *TFEB* gene
XBP1	[150]	In mouse liver, Xbp1 binds *Tfeb* promoter and regulates autophagy connected with metabolic functions
TF cooperating with TFEB in regulating gene expression		
CLOCK	[151]	TFEB directly interacts with the CLOCK/BMAL1 complex through CLOCK and regulates PER3 expression with circadian rhythm
FOXO	[152]	TFEB forms a heterocomplex
MITF	[5, 17, 18].	TFEB forms a heterocomplex
MONDOA	[153]	In HeLa cells, this TF favors TFEB nuclear translocation by an unknown mechanism. *Caenorhabidis elegans* ortholog MML‐1 exhibits similar behavior in the context of genetic program, sustaining longevity
NF‐κB	[136]	TFEB suppresses IκB kinase to protect IκBα from degradation, thereby, inhibiting NF‐κB p65 nuclear localization
PEG3	[133]	PEG3 is required for TFEB nuclear localization in endothelial cells stimulated with decorin, without showing any direct interaction
SMAD‐4	[100]	SMAD‐4 is required for TFEB expression induced by TGF‐β, but the specific mechanism was not molecularly addressed
STAT 3	[123]	TEFB indirectly upregulates STAT 3 in macrophages
TFC4	[154]	In glioblastoma, Wnt signal exploits TCF to regulate TFEB nuclear translocation though mTor‐mediated mechanism. TFC inhibition decreases mTor signaling
TFE3	[5, 17, 18, 71].	TFEB forms a heterocomplex
YY1	[155]	In melanomas, the heterocomplex TFEB/YY1 controls autophagic gene expression

^a^ TFEB recognizes binding sites on Ppargc promoter, which is a transcriptional co‐activator [69, 71].

Thirdly, continued characterization of the TFEB effect on stroma compartment of cancer might inform the generation of new approaches to improve the current stromal therapies based on immunotherapy and anti‐angiogenic regimens. The current knowledge of TFEB in regulating TME is in its infancy (Fig. 4) and new efforts are needed to investigate its role in other stroma components such as fibroblasts and nerves. These studies will reveal crucial feedforward and feedback circuits within cells belonging to TME that can influence cancer cell behavior that could benefit from specific TFEB regulation.

Fourthly, new findings of TFEB effects on key mechanisms of cancer progression including EMT, cell cycle, invasive properties and cancer stemness, may open up new translational opportunities.

## Conflict of interest

The authors declare no conflict of interest.

## Author contributions

EA wrote the sections on the general aspects of TFEB biology. FB inspired the review, wrote the sections on TFEB and cancer, and supervised the final version. GD wrote the sections on TME.
